# Virtual Reality Self-help Treatment for Aviophobia: Protocol for a Randomized Controlled Trial

**DOI:** 10.2196/22008

**Published:** 2021-04-12

**Authors:** Jamie Rhiannon Fehribach, Marieke Bianca Jolien Toffolo, Ilja Cornelisz, Chris van Klaveren, Annemieke van Straten, Jean-Louis van Gelder, Tara Donker

**Affiliations:** 1 Clinical Psychology Department of Clinical, Neuro- and Developmental Psychology Vrije Universiteit Amsterdam Amsterdam Netherlands; 2 Amsterdam Public Health Research Institute Amsterdam Netherlands; 3 Methods and Statistics Department of Education Sciences Vrije Universiteit Amsterdam Amsterdam Netherlands; 4 Amsterdam Center for Learning Analytics Amsterdam Netherlands; 5 Department of Criminology Max Planck Institute for the Study of Crime, Security and Law Freiburg Germany; 6 Institute of Education and Child Studies Faculty of Social and Behavioural Sciences Leiden University Leiden Netherlands; 7 Laboratory of Biological and Personality Psychology Department of Psychology Albert Ludwigs-University of Freiburg Freiburg Germany

**Keywords:** aviophobia, specific phobia, virtual reality, cognitive behavioral therapy, exposure therapy

## Abstract

**Background:**

Aviophobia (the fear of flying) can greatly impact the daily life functioning of people with the condition. Traditional exposure-based treatment is hampered by the limited availability of airplane practice situations, which is a result of economical and practical concerns. Easily accessible and low-cost virtual reality exposure therapy may address these challenges.

**Objective:**

The purpose of our study is to investigate the effectiveness of ZeroPhobia: Aviophobia (a self-help mobile app–based treatment) in reducing flight anxiety symptoms and depressive and anxiety symptoms. We will also investigate the effects of usage intensity, the sense of immersion, inherent absorption ability, and perceived user-friendliness on the treatment effect.

**Methods:**

Participants (N=114) who are aged 18-64 years and experience at least mild symptoms of aviophobia will be recruited from the general Dutch population and randomized into a treatment group or waitlist control group. By using their own phones and rudimentary mobile virtual reality headsets, participants will receive six modules of psychoeducation and cognitive behavioral therapy, which will include six levels of virtual reality exposure therapy over a period of 6 weeks. Assessments will be conducted at baseline, posttest (ie, after 6 weeks), and 3- and 12-month follow-ups. The primary outcome measure of our study is the Flight Anxiety Situations Questionnaire. The secondary outcome measures include anxiety and depression measures and additional covariates (including usage intensity, the degree of immersion, etc). We will test treatment effectiveness by conducting an intention-to-treat analysis and estimating average treatment effects on the treated. The mechanisms of treatment effect will also be explored.

**Results:**

The study was funded on September 25, 2018. Ethical approval was received on October 11, 2019. Recruitment closed on May 7, 2020.

**Conclusions:**

Our study will further the scientific understanding and clinical implications of technology’s current ability to aid in providing effective, accessible treatment for the fear of flying.

**Trial Registration:**

Netherlands Trial Registry NL70238.029.19; https://www.trialregister.nl/trial/8257.

**International Registered Report Identifier (IRRID):**

DERR1-10.2196/22008

## Introduction

Aviophobia, or the fear of flying, is one of the most common specific phobias. It has a lifetime prevalence rate of approximately 13.2% [1]. An estimated 10%-35% of people from Western Europe and North America experience enough flight anxiety that they avoid flying or do so with great discomfort and fear [2]. This avoidance, which is a common characteristic of specific phobia, not only perpetuates this fear, but also results in interruptions to one’s ability to live life. Being unable to visit family members abroad or having to quit a job that requires air travel are just two examples of how the fear of flying can impact an individual [3]. The high degree of aviophobia’s diffusive impact on day-to-day life is, in part, due to the heterogeneity of the phobia. Aviophobia stems from a range of subfears and reasons for fear [2]. Anxiety toward flying can be rooted in a fear of heights (acrophobia) or a fear of small or inescapable spaces (claustrophobia). Such anxiety can also result from fears of a plane crashing or the inability to have control over a flight experience [2]. Aviophobia, as well as other specific phobias, stands the risk of becoming chronic if left untreated. It is also connected with the comorbid development of other mental health problems [4]. Therefore, effective, accessible, and affordable treatments are necessary.

Specific phobia is most commonly treated via cognitive behavioral therapy with exposure therapy [5]. During exposure therapy, a person is gradually exposed to their feared stimuli, starting from their least to most feared stimulus. Exposure therapy is most often conducted face-to-face with a therapist in real-world (or in vivo) environments [6]. This design however gives rise to several challenges, especially when it comes to aviophobia. For instance, (1) monetary and temporal costs are high due to the need for a therapist and real-world materials for conducting an exposure (eg, plane tickets, time for traveling to and from the airport or flight destination, etc) [7,8]; (2) waitlists are longer, again due to the need for a therapist over the course of treatment [9]; and (3) many individuals are too afraid to begin exposure therapy or drop out before the therapy is complete due to fears of exposures [10].

Researchers have already begun to search for new ways to approach these challenges, including the implementation of virtual reality (VR) technology. Unlike traditional methods, VR exposure therapy (VRET) uses computer-generated environments for exposure, thus addressing the costs of travel and stimuli for exposures. Additionally, research has shown that individuals are less averse to beginning VRET and more averse to beginning in vivo treatment due to a fear of confronting feared stimuli in real life [11].

Several studies on the fear of flying that use VRET have demonstrated positive results [12,13]. A meta-analysis found that VRET for the fear of flying was superior to control conditions (*g*=1.35; *P*<.001) and classical evidence-based treatments (*g*=0.35; *P*=.01) that included interventions that are used to conduct in vivo exposure therapy [14]. Therefore, VRET for the fear of flying is a viable alternative to in vivo treatment.

Although VRET addresses many of the challenges in treating patients with aviophobia, most treatments still involve a therapist to guide clients through the program [15]. This means that waitlists for available therapists still impede people who seek treatment. Several VRET studies have demonstrated the potential of technological treatments to be effectively self-guided [16-18]. In these VRET studies, participants followed treatment programs without a guiding therapist. However, they were still required to complete sessions in a lab with an assistant present, making these studies’ results less reflective of real-life experiences.

ZeroPhobia is a VRET program that was designed to investigate whether VRET can in fact be completed at home without a therapist and with basic equipment. This program only requires the user to have a compatible phone (ie, one that contains a gyroscope) and a mobile VR headset, thus reducing the severity of the aforementioned treatment barriers (temporal and monetary costs, waitlists for an available therapist, and aversion to facing real-life stimuli). Furthermore, it has been reported that ZeroPhobia is effective in treating acrophobia, with an effect size (*d*) of 1.14 (intention-to-treat [ITT] group compared to a waitlist group) [15].

The primary aim of our study is to investigate whether ZeroPhobia: Aviophobia (a self-help mobile app–based treatment) can effectively reduce flight anxiety symptoms by using the most basic and affordable VR equipment (ie, a mobile VR headset and participants’ own smartphones) and whether these improvements can be maintained in the long term. This will be confirmed by analyzing changes in flight usage between the pretest and posttest periods and at follow-up time points. We will also test the effects of ZeroPhobia on anxiety and depressive symptoms. Additionally, we aim to investigate whether ZeroPhobia: Aviophobia is user-friendly, to measure app usage intensity, and to determine whether participants felt present and immersed in VR.

Although VR technology’s ability to induce feelings of “being there” (presence) through immersive technology is well known, studies have found conflicting evidence on the importance of presence for successful VRET [8,19-24]. One factor that could influence this relationship is a person’s inherent ability to feel like they have been transported into a fantasy environment. This feeling is known as absorption [24,25]. Studies have suggested that during VRET with low-immersion technologies, an individual’s degree of absorption is related to the efficacy of the treatment [26]. Since ZeroPhobia uses relatively low-immersion technology, absorption may be an important mediating factor for our outcome. Therefore, absorption will be explored.

We hypothesize that participants from the general Dutch population who are randomized into the treatment group will experience a greater reduction in flight anxiety symptom severity between the pretest and posttest period compared to those in the waitlist control group. We also hypothesize that this reduction in flight anxiety symptom severity will be maintained at the 3- and 12-month follow-ups. Additionally, we hypothesize that individuals will experience less severe general anxiety and depression after treatment. We further hypothesize that ZeroPhobia: Aviophobia will be rated as user-friendly and that participants will feel immersed in VR environments. Finally, we hypothesize that higher usage intensity, a higher sense of presence, higher inherent absorption ability, and higher perceived user-friendliness will be associated with a reduction in flight anxiety symptom severity at posttest.

## Methods

### Study Design

Our study is a randomized controlled trial with a superiority design. We will compare the following two conditions: the intervention and waitlist control conditions. The duration of both the treatment intervention and waitlist waiting periods is 6 weeks. After the waiting period, participants who were randomized into the control group will receive 6 weeks of treatment.

**Figure 1:**
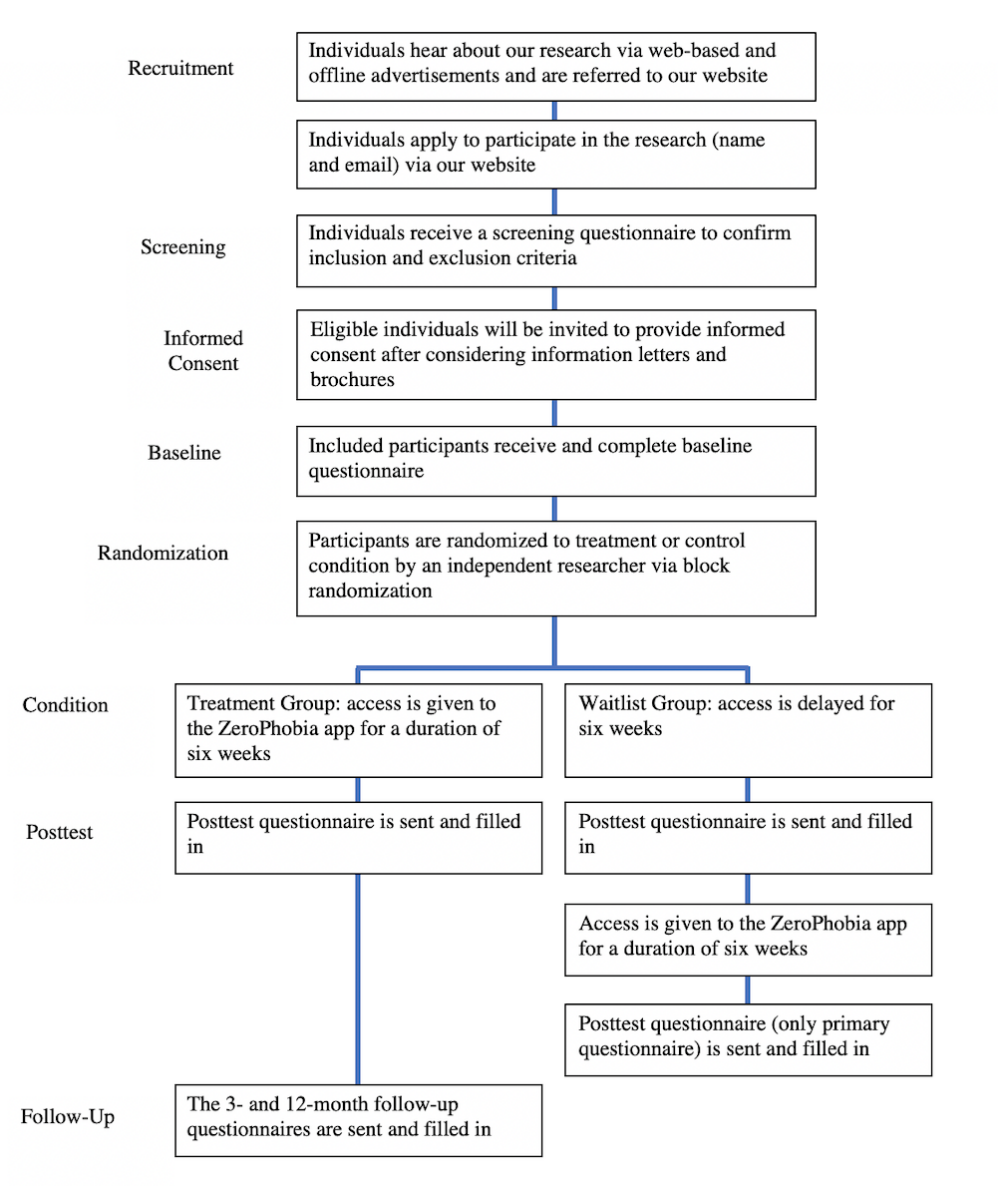
Participant flowchart.

A total of 114 participants will be recruited, and each condition group will include 57 participants. Further details are provided in the “Sample Size” section of this paper. Participants in the intervention group will complete web-based questionnaires at baseline, posttest, and the 3- and 12-month follow-up time points. Participants in the waitlist control group will complete web-based questionnaires at baseline, at posttest, and after receiving the intervention treatment. Figure 1 shows a participant flowchart. Multimedia Appendix 1 includes a SPIRIT (Standard Protocol Items: Recommendations for Interventional Trials) flow diagram.

Our study received ethical approval from the Medical Ethical Committee of Vrije Universiteit Medical Center (registration number: 2019-321). The study is registered in the Netherlands Trial Registry (trial number: NL70238.029.19).

### Procedure

Participants will be recruited from the general Dutch population via advertisements on web-based platforms (such as Facebook, Instagram, and webpages that belong to the following fear of flying courses: Stichting Valk and Onbezorgd Vliegen) and offline platforms (ie, national radio, newspaper/magazine articles, and flyers). These advertisements will direct interested individuals to the study website, on which detailed information about the study is available. Those who are interested and believe that they are eligible will be directed to complete a brief web-based response form to express their interest. No incentives were offered to participants other than the opportunity to receive the novel treatment as part of our research.

The research team will then respond to interested individuals by providing additional informational materials and a web-based screening questionnaire to determine eligibility. Those who complete our screening questionnaire but are deemed ineligible will receive an automated email response informing them of why they are unable to participate in the study. Potential participants will be reminded via email twice to complete the screener; if potential participants do not provide a response or are no longer interested, they will be removed from our contact list.

Those who are found to be eligible for the study will receive an informed consent form and participant information letter with an enclosed preaddressed return envelope via mail. Participants will receive two reminders (first by email, then by phone) to return the signed consent form. This consent form will ask participants to consider whether they would like to receive information on the results of the overall study, which we will share after the analysis is complete. After returning their signed consent form via mail or email, participants will be provided with our baseline questionnaire. After completing this questionnaire, participants will be included in the study, and an independent researcher will randomly assign participants to either the treatment group or waitlist group by using the Random Allocation Software program (MathWorks). Participants will be informed of which group they are assigned to; those randomized into the waitlist group will be told that they will be contacted again in 6 weeks, and those randomized into the treatment group will be provided with a mobile VR headset and usage instructions via mail. Participants will also be provided with instructions on how to download and install the ZeroPhobia app and a unique unlock code to access the app.

After downloading the app, participants will be able to freely use the ZeroPhobia app at their own pace. The app includes 6 modules that participants can complete. Weekly emails will be sent to remind participants to continue using ZeroPhobia during the treatment period. These emails will be sent out regardless of how much time a participant has spent on using the app. Weekly surveys that measure flight anxiety were not included in this procedure. This allows us to use the most naturalistic approach of applying the intervention and to lighten the burden on an individual. In the final module, participants will be encouraged to engage in real-world exposures (ie, booking an airplane flight or visiting airports). After 6 weeks, participants from both groups will receive an invitation to complete a posttest questionnaire.

Once posttest questionnaires are completed, intervention group participants will be told that they will be contacted again after 3 and 12 months and that they are free to continue using ZeroPhobia if they wish. Control group participants will be invited to begin using ZeroPhobia. During the intervention period, they will also receive weekly reminders. After 6 weeks of ZeroPhobia use, participants will be asked to complete one final questionnaire that only pertains to the primary outcome measure.

### Inclusion and Exclusion Criteria

To be included in the study, participants must achieve a score of ≥56 on the Dutch version of the Flight Anxiety Situations Questionnaire (FAS), which indicates mild flight anxiety [27,28]. Additional inclusion criteria include an age of between 18 and 64 years, the ability to speak proficient Dutch, the possession of a compatible smart phone (either an iPhone [version 5 or higher] or an Android phone [that operates on Lollipop version 5.1 or higher with screen sizes of 4.7-5.5 inches] with access to the internet), and the provision of informed consent to participate. Exclusion criteria include receiving other psychological treatments for aviophobia at the time of the study or starting, adjusting, or planning to start or adjust psychotropic medication from 3 months prior to the time of recruitment to the planned end time of the study.

### Sample Size

Our primary outcome measure of aviophobia, the FAS, was used for power calculations. Previous meta-analytical studies on VRET for the fear of flying have found that such therapy has an effect size (Cohen *d*) of 0.80 [29]. Since ZeroPhobia: Aviophobia is a self-help treatment that involves rudimentary VR glasses instead of standard VRET with a therapist, we used a slightly more conservative effect size (*d*=0.70) as the starting point for our power calculations. To identify a difference between the experimental and control conditions with a standardized effect size of 0.70 (determined with a two-tailed *t* test for assessing differences between two independent means), an α value of .05, and a statistical power (1−β) of .80, we require 68 respondents (34 respondents per group). After taking into consideration an expected dropout rate of 40% [15], our overall required sample size is 114 (57 participants per group).

### Randomization, Blinding, and Treatment Allocation

Randomization will be conducted with the Random Allocation Software program, which uses the block randomization method for conducting a 1:1 allocation and random block sizes of 6, 8, 10, and 12. Randomization will be conducted by an independent researcher. This researcher will generate a randomization list (a list of outcomes) that will be used to assign participants to either the treatment group or waitlist group. The blinding of participants and the research team to who is assigned to each group is not possible due to the nature of our study. However, all measurements will be completed by the participants via a web-based platform, without the presence of the research team.

### Intervention

ZeroPhobia: Aviophobia is an app-based intervention that consists of six modules that can be completed at the user’s own pace. ZeroPhobia was created with aesthetic in mind, making the graphics and content visually pleasing and logically designed. There is no guide or therapist involved, only a preprogrammed virtual therapist who explains how to use the app and educates the user. Therapeutic content provided by the modules was created by following the manualized protocols of Cognitive Behavioral Therapy for Specific Phobias [30-32]. The app was designed for smartphone use only, because the device must be small enough to double as the screen for VR glasses during VRET.

Each module takes 5-20 minutes to complete, depending on how fast the user wishes to go through a module. Cognitive behavioral therapy content is provided by the virtual therapist and the use simple, 2D animations and voice overs. Module 1 (Achtergrond/Background) describes what flight anxiety is and facts about airplane safety. In this module, participants are also introduced to two faux past ZeroPhobia users, Chloe and Bruno. Both appear across modules in order to share their experiences with ZeroPhobia and offer examples of how flight anxiety develops and how it is overcome, thus further educating participants. Module 2 (Je angst te lijf/Facing Your Fears) teaches participants about the anxiety curve and how to create realistic goals to overcome fears of flying.

Module 3 explains how to effectively and safely undergo VRET. After completing this module, the VR levels become unlocked. These levels include detailed 3D animations of the following flight scenarios: checking in at an airport, boarding a plane, finding a seat, taking off, flying, flying with turbulence, and landing. Each of these scenarios involves games, such as finding objects or comforting other nervous passengers, that are led by the virtual therapist. These games help to increase engagement. Participants are able to unlock and progress through VR levels by rating their anxiety with a score of ≤3. Scores are based on a scale of 1-10, with 1 indicating very little anxiety. If a participant completes a VR level and rates their anxiety during the level with a score of >3, a message appears. This message explains that they should practice this level again before moving on to the next level.

Participants will be encouraged to practice with the VRET levels 10 minutes per day and the final three final modules once per week. Before and after finishing a VRET level, participants are prompted to rate their anxiety (“How high was your anxiety at its peak during this level?”). Participants will receive preprogrammed feedback on this rating, which will advise them to either move on to the next VRET level or repeat the level again until their anxiety is lower.

**Figure 2:**
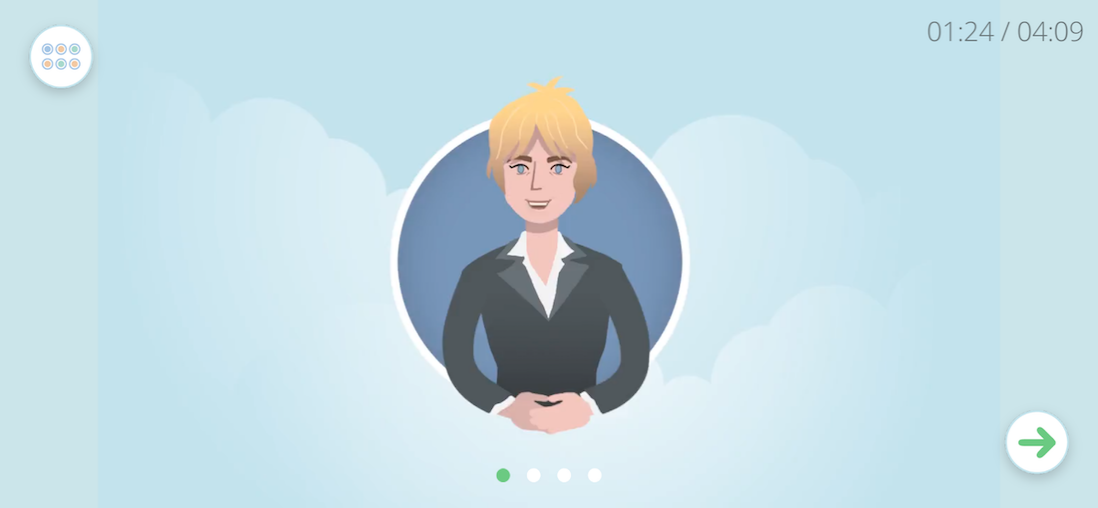
The virtual therapist and guide of ZeroPhobia.

**Figure 3:**
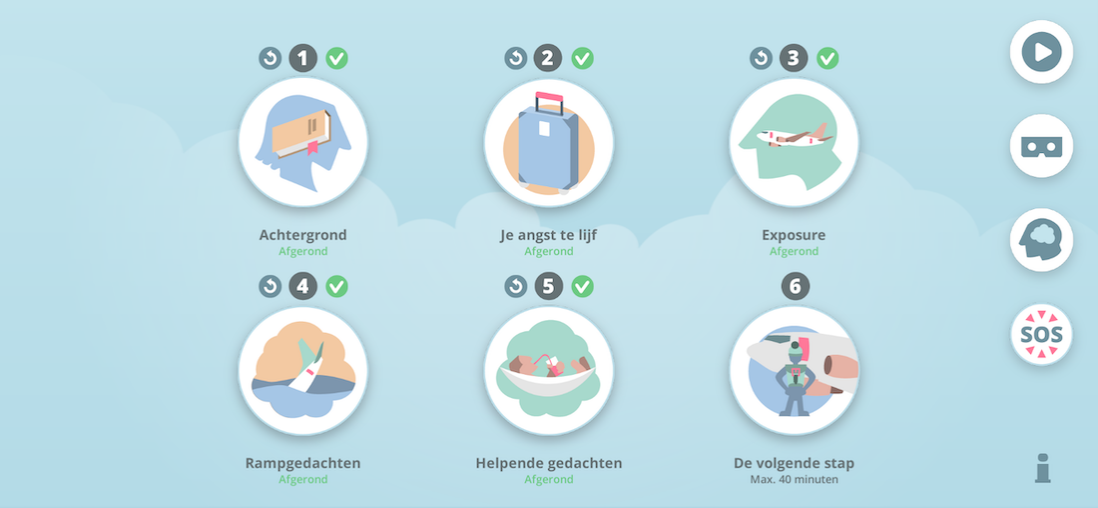
The home screen of ZeroPhobia. For translations of module names, see Table 1.

**Figure 4:**
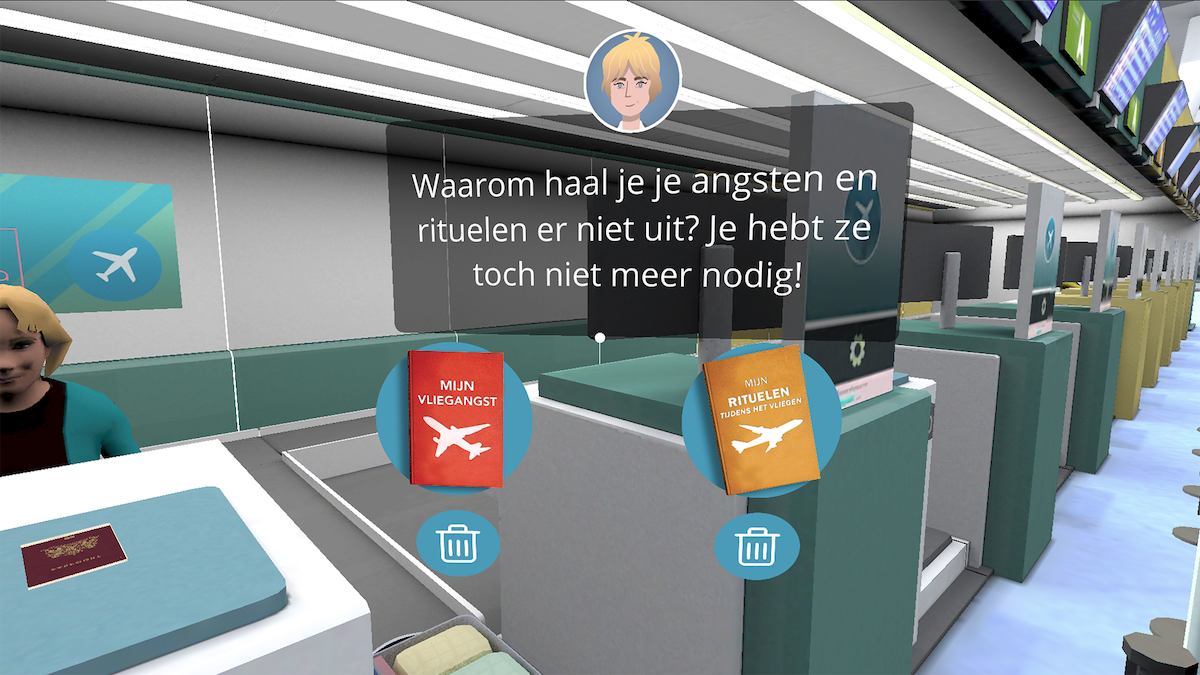
Virtual Reality Level 1: Checking In. The text in the therapist's speech bubble translates to “Why don’t you take your fears and rituals out (of your suitcase)? You don’t need them anymore!” The text on the red book translates to “My Flight Anxiety”. The text in the yellow book translates to “My Rituals During Flights”.

**Figure 5:**
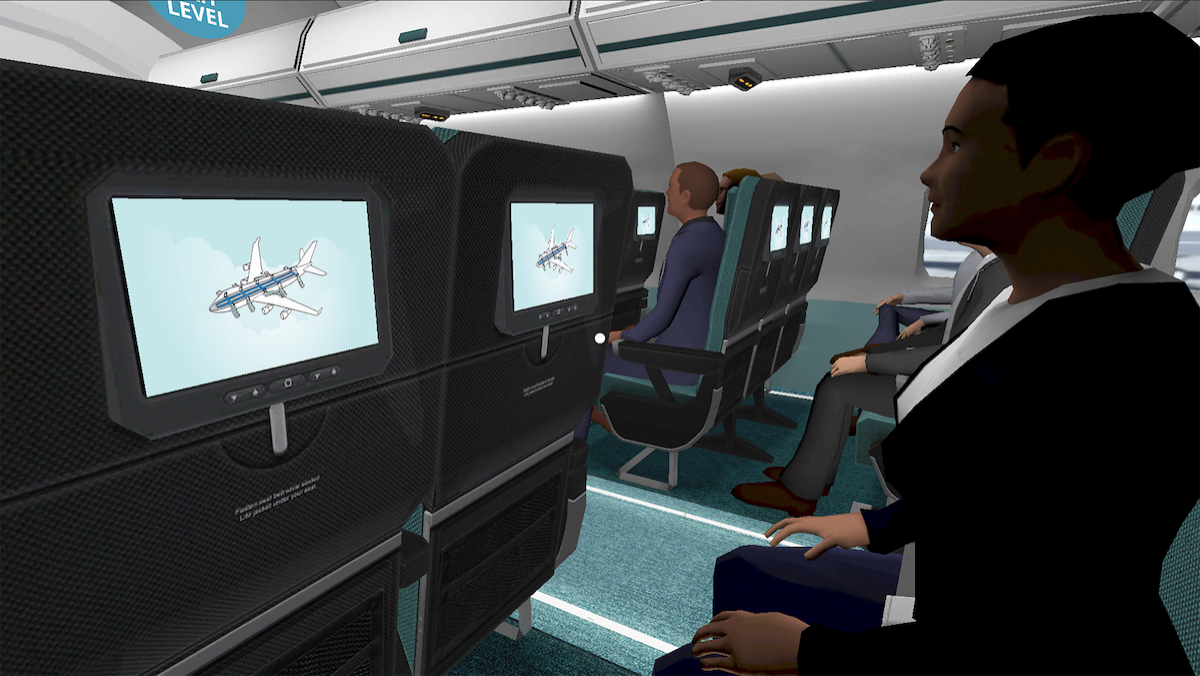
Virtual Reality Level 4: Taking Off.

Participants will be encouraged to practice VRET as they complete the final three modules. Module 4 focuses on educating participants about automatic, catastrophic thoughts and identifying their own thoughts. Module 5 expands on this topic by teaching participants about helping thoughts that they can construct to combat catastrophic thinking. Finally, module 6 encourages participants to use their newfound knowledge and begin to practice exposure in the real world. Participants will continue to have access to ZeroPhobia after the test phase is completed and during follow-up. Table 1 provides an overview of all modules, and Figures 2-5 show images of the ZeroPhobia app.

**Table 1:** Module overview.

Module	Learning objective	Additional information
Module 1: Achtergrond/Background	Understand what the fear of flying is and how it develops	Participants are introduced to two fictional characters who have also followed ZeroPhobia. These characters will provide further examples.
Module 2: Je angst te lijf/Facing Your Fears	Create goals and learn about the anxiety curve	Participants create goals such as “I would like to be able to take a short flight in a few months with less anxiety than I feel now.”
Module 3: Exposure	Learn about exposure, the completion of exposure levels, and safety information	After completing this level, the first virtual reality level is unlocked. In order to continue unlocking VR levels, participants must report low levels of anxiety (ie, a score of ≤3 on a scale of 1-10) for the current level.
Module 4: Rampgedachten/Catastrophic Thoughts	Learn about what automatic, catastrophic thoughts are and how to identify such thoughts (ie, their own thoughts)	Participants learn about how automatic, catastrophic thoughts can increase and maintain anxiety. Participants are also encouraged to reflect on how realistic their catastrophic thoughts actually are.
Module 5: Helpende gedachten/Helping Thoughts	Formulate helping thoughts to combat catastrophic thoughts	Participants must think of reasons for why their catastrophic thoughts are not realistic and the unrealistic thoughts they have about their fears.
Module 6: De volgende stap/The Next Step	Become inspired to begin practicing in vivo exposures and formulate a fear hierarchy	An example fear hierarchy is created by using one of the fictional characters, who provide recommendations on how to reward oneself for completing different fear hierarchy challenges.

### Primary Outcome Assessment (FAS)

The FAS [27] is a Dutch, 32-item, self-report questionnaire that is frequently used to assess the fear of flying. The response for each item is based on a 5-point Likert scale (1=no fear; 5=overwhelming fear). The questionnaire focuses on measuring how much anxiety is induced by different airport or flight situations. The reliability of the FAS ranges from good to excellent, and the questionnaire is sensitive in distinguishing between people with and without the fear of flying [28]. The total possible score on this measure ranges between 32 and 160. This measure will be used for screening purposes; it has a cutoff score of 56, which indicates mild anxiety [23,24]. The FAS will be used again at posttest and during both follow-up time points.

### Secondary Outcome Assessment

#### Flight Anxiety Modality Questionnaire

The Flight Anxiety Modality Questionnaire (FAM) [27] is a Dutch, 18-item, self-report questionnaire. FAM scores are a secondary measurement to FAS scores; they are used to maintain the robustness of results. Similar to the FAS, responses to FAM items are based on a 5-point Likert scale that ranges from 1 (not at all) to 5 (very much so). The reliability of this questionnaire has also been found to be good [28]. Unlike the FAS however, the FAM focuses on the degree to which different anxiety symptoms are experienced in flight situations. This measure will be assessed at baseline, at posttest, and during both follow-up time points.

#### Beck Anxiety Inventory

The Beck Anxiety Inventory [33] is a self-report questionnaire that consists of 21 items on a 4-point Likert scale, which ranges from 0 (not at all) to 3 (severely; “I could barely stand it”). The total possible score ranges from 0 to 63. Scores of 0-9 indicate no anxiety, scores of 10-18 indicate mild to moderate anxiety, scores of 19-29 indicate moderate to severe anxiety, and scores of 30-36 indicate severe anxiety. The questionnaire’s internal consistency is high (0.90-0.94) and its validity is good [34]. For our study, the Dutch translation of the Beck Anxiety Inventory will be used [35]. This measure will be assessed at baseline, at posttest, and during both follow-up time points.

#### Patient Health Questionnaire

The Dutch translation [36] of the 9-item Patient Health Questionnaire [37] will be used to assess depressive symptoms at baseline, at posttest, and during follow-up time points. This questionnaire has good sensitivity (0.71-0.84) and specificity (0.90-0.97) [38].

#### Web Screening Questionnaire

The Dutch Web Screening Questionnaire [39] consists of 15 items and screens for an array of common mental health disorders (eg, depressive disorder, alcohol abuse/dependence, etc). For the purposes of our study, only items that assess panic disorder, agoraphobia, and obsessive-compulsive disorder will be used, as these disorders involve symptoms that overlap with those of flight anxiety. The Web Screening Questionnaire has good sensitivity (0.72-1.00) and specificity (0.44-0.77) [39]. This questionnaire will only be provided at baseline to assess whether the presence of panic disorder, agoraphobia, or obsessive-compulsive disorder influences posttest flight anxiety outcomes.

#### Credibility/Expectancy Questionnaire

The 6-item Credibility/Expectancy Questionnaire (CEQ) [40] will be used to assess self-reported treatment expectations at baseline. The CEQ has high internal consistency and good test-retest reliability [40]. Studies have also found that the Dutch translation of the CEQ is valid [41]. This questionnaire will be used as a complement to the Client Satisfaction Questionnaire (CSQ) to better understand whether the treatment met participants’ expectations.

#### CSQ Assessment

The CSQ [42] complements the CEQ. It measures participants’ satisfaction with ZeroPhobia at posttest. This questionnaire consists of 8 items that assess whether ZeroPhobia fulfilled participants’ expectations and whether participants would recommend Zerophobia: Aviophobia treatment to someone else with similar flight anxiety symptoms. Studies have found that the CSQ is both valid and internally reliable (Cronbach α values range between .83 and .93) [39]. The Dutch translation has also been tested and found to be valid [43].

#### System Usability Scale

The System Usability Scale (SUS) [44] consists of 10 items that measure the user-friendliness of the ZeroPhobia app. The response to each item is based on a 5-point Likert scale (1=not in agreement; 5=very much in agreement). Total scores are calculated and converted so that they range from 0 to 100. Bangor et al [44] have provided details on this conversion. Higher scores indicate better usability and higher user-friendliness. SUS items include “I think I would gladly use the ZeroPhobia app regularly” and “I found the ZeroPhobia app unnecessarily complex.” In order for a system to be considered passable, it must receive a score of ≥70. Better products receive scores of ≥80. Only incredibly high–quality products are expected to receive a score of >90. Systems that receive a score of <70 are considered inadequate and should be reassessed and improved. The reliability of the SUS is good [44], and the validity of Dutch translation of the SUS is comparable to that of the original measure [45]. The SUS questionnaire will only be provided (at posttest) to the intervention group.

#### Igroup Presence Questionnaire

The 14-item Igroup Presence Questionnaire [19] assesses the realism of VR environments and the feeling of “presence” or immersion in VR environments. Responses to each questionnaire item is based on a 7-point Likert scale that ranges from −3 (fully disagree) to 3 (fully agree). The questionnaire includes items such as “I had the feeling of being surrounded by the virtual world.” This questionnaire has been found to be reliable (Cronbach α=.73) [19]. Schubert et al [19] translated this questionnaire from German to both Dutch and English; the Dutch version will be used for our study. This questionnaire will only be provided (at posttest) to the treatment group.

#### Questions on Real-Life Flight Usage

Single-item questions will be asked at baseline, at posttest, and during both follow-up time points to understand changes in flight usage during the study. Baseline questions will ask participants about whether they have ever flown, how many flights they have taken in the past year, and how long each of these flights lasted. The questions asked during subsequent time points will ask participants to provide similar information. These questions will be adjusted according to the study phase. For instance, at posttest, participants will be asked “how many flights have you taken in the past six weeks?” These questions were formulated for the purposes of our study.

#### Ecological Momentary Assessment

Directly after each VRET session, the app will prompt participants to rate how much anxiety (ie, peak anxiety) they experienced during a level on a scale of 1-10. These ratings will be used to further our understanding of the efficiency of our VR environments and advise a participant to either move on to the next VR environment or to practice again. For example, an anxiety rating of ≥4 indicates that participants are still experiencing moderate to high anxiety during a level.

### Additional Measures

#### Interpersonal Reactivity Index

The Interpersonal Reactivity Index (IRI) [46] is a self-report questionnaire that was created to measure different types of empathy. The Fantasy subsection of the IRI will be used at baseline to understand the general fantasizing abilities of participants. The IRI-Fantasy subscale consists of 7 items on a 5-point Likert scale (0=this does not describe me; 4=this describes me very well). By using this questionnaire, we will be able to investigate a person’s ability to experience emotional absorption or feelings of being transported into a fantasy world. This questionnaire has been found to be both reliable and valid [46]. The Dutch translation of this scale has also been found to be reliable and valid [47].

#### Questions on Professional Treatment

Single-item questions regarding other psychotherapeutic treatments that participants are undergoing during the study (either psychotherapy or prescriptions for psychotropic medication) will be asked during screening, at posttest, and during both follow-up time points. These questions will be asked to ensure that if a participant does start a different treatment, we can account for this properly in our analysis. Table 1 provides an overview of which assessments are conducted for each time point.

### Analyses

#### Covariates and Sample Differences

Analyses will be conducted with Stata 16 (StataCorp LLC). First, the external validity and generalizability of the experimental sample will be determined by comparing it to the overall sample. Second, we will validate whether randomization was successful by examining whether there are significant differences in background characteristics between the intervention and control groups. To check for selective attrition, we will show how missing data (outcome and input data) are divided across experimental groups. To keep the experimental sample intact, a dummy variable will be constructed to represent the missing covariates of a participant. Missing covariate values will be replaced with their mean values.

#### Estimating Treatment Effect

The treatment effect will be estimated by means of ordinary least squares regression, which will be based on an intention-to-treat (ITT) analysis that uses treatment assignment status as the principal predictor. Missing outcome observations for participants will be imputed by using multiple imputation methods for assessing pretest scores and a set of prespecified background characteristics (gender, age, education, and the severity of symptoms).

Nonrandom missing outcome observations can result in biased point estimates; a treatment effect estimate that is obtained via complete case analysis or the estimation of ITT effects may be a biased estimate of the average treatment effects on the treated (ATT). This issue will be addressed via the Random Forest Lee Bounds procedure [48], which is used to trim the outcome distribution of the group with the highest attrition toward the group with the lowest attrition. By removing observations from the lower (upper) end of the distribution, the lower (upper) bound of the ATT can be estimated via ordinary least squares regression. The potentially biased ATT point-estimate will then lie within the estimated treatment effect bounds.

The ATT will be estimated and corresponding measures of clinically meaningful changes and values in treatment effects on the treated will be explored.

#### Mechanisms of Treatment

Underlying mechanisms of observed differences will be investigated via analyses for understanding factors such as user-friendliness, treatment expectation and satisfaction, usage intensity, and feelings of absorption and presence. The robustness and sensitivity of these analyses will be tested by using different assumptions based on the data.

#### Data Monitoring and Management

All data will be collected by researchers from the Clinical Psychology Department of Vrije Universiteit Amsterdam and kept confidential in accordance with the General Data Protection Regulation. The results of our trial will be released regardless of whether they are statistically significant in terms of answering our research questions. This is in line with the publication statement of the Dutch human research commission (Centrale Commissie Mensgebonden Onderzoek).

#### Risk to Participants

Past research has shown that VRET can be administered without introducing significant safety risks to participants [21,48-51]. Additionally, previous research on the ZeroPhobia app has found that using the app does not result in serious adverse effects (no participants reported that they acquired injuries during VR use or experienced unbearable anxiety levels while using the app) [15].

### Protocol Amendments

If amendments are made to the protocol as it is written now, these will be reflected in the trial registry after receiving approval from the Medical Ethical Committee. The results of our study will be published in peer-reviewed journals once they are available.

## Results

Our study received funding on September 25, 2018. Ethical approval was obtained on October 11, 2019. Data collection began on November 6, 2019. Recruitment closed on May 7, 2020, and 146 participants were recruited. On July 2020, participants were in the final stages of treatment or in the 3- or 12-month follow-up phase. Data collection is projected to be completed in July 2021.

## Discussion

In our study, we aim to understand the effectiveness of our self-help app, Zerophobia: Aviophobia, in reducing flight anxiety symptoms. Traditional treatment for the fear of flying is often not accessible to many patients due to the high costs of both the therapists and the materials (ie, plane tickets) that are necessary for in vivo treatment [7,8]. Moreover, many patients are generally reluctant to face their fear and begin in vivo exposure therapy [11]. Untreated specific phobia is related to a high risk of developing anxiety and depression [4], which increase the overall negative impacts of specific phobia and the need for an easily implemented treatment.

Over the past decade, VRET has become a viable alternative to traditional in vivo treatment, as evidenced by VRET’s effectiveness, which is comparable to that of in vivo treatment [14]. The evidence on the potential of automated or unguided self-help VRET for phobias also shows that there is a need to further investigate VRET [16,17]. Our study will provide novel insights into automated VRET and methods for making the treatment of specific phobia more accessible to the general population.

Approximately 60%-80% of individuals with specific phobias do not seek treatment for such anxiety disorders, and approximately 25% of individuals with specific phobias who do seek treatment refuse to undergo treatment due to a fear of the therapeutic procedures [11]. With regard to people with aviophobia, those who are interested in receiving treatment face accessibility issues, which are primarily the result of the high costs in acquiring exposure materials (eg, paying for a plane ticket). Our study is therefore specifically aimed at patients who would normally not receive any treatment. As such, our study compares patients who undergo a novel VRET intervention against patients in a waitlist control group. This design is in line with those of other studies that show that a control group is comparable to intervention groups that undergo treatment as usual for a specific phobia [16]. However, we have included measures for the uptake of all interventions/cointerventions in the treatment and control groups. In future studies, it would be interesting to compare the effectiveness/cost-effectiveness of regular therapy directly to that of our new VR therapy.

In summary, our study will assess whether an unguided self-help VR app that uses rudimentary VR glasses will be effective in reducing the severity of people’s fear of flying. We will also investigate whether symptoms of depression and anxiety are influenced by Zerophobia: Aviophobia treatment. Additionally, our study will determine whether the user-friendliness of similar apps, the degree of immersion in VR environments, people’s inherent absorption abilities, or usage intensity influence the effectiveness of VRET. Finally, we will assess whether these effects can be maintained in the long term (ie, 3 and 12 months following intervention).

## Appendix

Multimedia Appendix 1 SPIRIT (Standard Protocol Items: Recommendations for Interventional Trials) flow diagram. Only participants who were randomized into the treatment group were assessed with these measures.

## Abbreviations

ATT: average treatment effects on the treated
CEQ: Credibility/Expectancy Questionnaire
CSQ: Client Satisfaction Questionnaire
FAM: Flight Anxiety Modality Questionnaire
FAS: Flight Anxiety Situations Questionnaire
IRI: Interpersonal Reactivity Index
ITT: intention-to-treat
SPIRIT: Standard Protocol Items: Recommendations for Interventional Trials
SUS: System Usability Scale
VALK: VliegAngstbestrijding Leidse universiteit, Koninklijke Luchtvaart Maatschappij Naamloze vennootschap
VR: virtual reality
VRET: virtual reality exposure therapy

## References

[1] Curtis G, Magee WJ, Eaton WW, Wittchen H, Kessler RC (2018). Specific fears and phobias. Br J Psychiatry.

[2] Oakes M, Bor R (2010). The psychology of fear of flying (part I): a critical evaluation of current perspectives on the nature, prevalence and etiology of fear of flying. Travel Med Infect Dis.

[3] Foreman E, Bor R, van GL (2006). The nature, characteristics, impact and personal implications of fear of flying. Aviation Mental Health: Psychological implications for Air Transportation.

[4] Trumpf J, Margraf J, Vriends N, Meyer AH, Becker ES (2010). Specific phobia predicts psychopathology in young women. Soc Psychiatry Psychiatr Epidemiol.

[5] Abramowitz JS (2013). The practice of exposure therapy: relevance of cognitive-behavioral theory and extinction theory. Behav Ther.

[6] Wolitzky-Taylor KB, Horowitz JD, Powers MB, Telch MJ (2008). Psychological approaches in the treatment of specific phobias: a meta-analysis. Clin Psychol Rev.

[7] Rothbaum BO, Hodges L, Smith S, Lee JH, Price L (2000). A controlled study of virtual reality exposure therapy for the fear of flying. J Consult Clin Psychol.

[8] Rothbaum BO, Anderson P, Zimand E, Hodges L, Lang D, Wilson J (2006). Virtual reality exposure therapy and standard (in vivo) exposure therapy in the treatment of fear of flying. Behav Ther.

[9] Kazdin A (2013). Moderators, mediators and mechanisms of change in psychotherapy. Quantitative and Qualitative Methods in Psychotherapy Research.

[10] Olatunji BO, Deacon BJ, Abramowitz JS (2009). The Cruelest Cure? Ethical Issues in the Implementation of Exposure-Based Treatments. Cogn Behav Pract.

[11] Garcia-Palacios A, Botella C, Hoffman H, Fabregat S (2007). Comparing acceptance and refusal rates of virtual reality exposure vs. in vivo exposure by patients with specific phobias. Cyberpsychol Behav.

[12] Krijn M, Emmelkamp PMG, Olafsson RP, Bouwman M, van Gerwen LJ, Spinhoven P, Schuemie MJ, van der Mast CAPG (2007). Fear of flying treatment methods: virtual reality exposure vs. cognitive behavioral therapy. Aviat Space Environ Med.

[13] Tortella-Feliu M, Botella C, Llabrés J, Bretón-López JM, del Amo AR, Baños RM, Gelabert JM (2011). Virtual reality versus computer-aided exposure treatments for fear of flying. Behav Modif.

[14] Cardoş RAI, David OA, David DO (2017). Virtual reality exposure therapy in flight anxiety: A quantitative meta-analysis. Comput Human Behav.

[15] Donker T, Cornelisz I, van Klaveren C, van Straten A, Carlbring P, Cuijpers P, van Gelder J (2019). Effectiveness of self-guided app-based rirtual reality cognitive behavior therapy for acrophobia: A randomized clinical trial. JAMA Psychiatry.

[16] Freeman D, Haselton P, Freeman J, Spanlang B, Kishore S, Albery E, Denne M, Brown P, Slater M, Nickless A (2018). Automated psychological therapy using immersive virtual reality for treatment of fear of heights: a single-blind, parallel-group, randomised controlled trial. Lancet Psychiatry.

[17] Hong Y, Kim HE, Jung YH, Kyeong S, Kim JJ (2017). Usefulness of the mobile virtual reality self-training for overcoming a fear of heights. Cyberpsychol Behav Soc Netw.

[18] van Gerwen LJ, Koopmans TA (2018). Self-help treatment for fear of flying. Aeronautics and Aerospace Open Access Journal.

[19] Schubert T, Friedmann F, Regenbrecht H (2001). The experience of presence: Factor analytic insights. Presence (Camb).

[20] Slater M, Wilbur S (1997). A framework for immersive virtual environments (FIVE): Speculations on the role of presence in virtual environments. Presence (Camb).

[21] Benbow AA, Anderson PL (2019). A meta-analytic examination of attrition in virtual reality exposure therapy for anxiety disorders. J Anxiety Disord.

[22] Pertaub DP, Slater M, Barker C (2002). An experiment on public speaking anxiety in response to three different types of virtual audience. Presence (Camb).

[23] Krijn M, Emmelkamp PMG, Olafsson RP, Biemond R (2004). Virtual reality exposure therapy of anxiety disorders: a review. Clin Psychol Rev.

[24] Gromer D, Reinke M, Christner I, Pauli P (2019). Causal interactive links between presence and fear in virtual reality height exposure. Front Psychol.

[25] Bissonnette J, Dubé F, Provencher MD, Moreno Sala MT (2016). Evolution of music performance anxiety and quality of performance during virtual reality exposure training. Virtual Real.

[26] Ling Y, Nefs HT, Morina N, Heynderickx I, Brinkman WP (2014). A meta-analysis on the relationship between self-reported presence and anxiety in virtual reality exposure therapy for anxiety disorders. PLoS One.

[27] Van Gerwen LJ, Spinhoven P, Van Dyck R, Diekstra RFW (1999). Construction and psychometric characteristics of two self-report questionnaires for the assessment of fear of flying. Psychol Assess.

[28] Nousi A, van Gerwen L, Spinhoven P (2008). The Flight Anxiety Situations Questionnaire and the Flight Anxiety Modality Questionnaire: norms for people with fear of flying. Travel Med Infect Dis.

[29] Fodor LA, Coteț CD, Cuijpers P, Szamoskozi S, David D, Cristea IA (2018). The effectiveness of virtual reality based interventions for symptoms of anxiety and depression: A meta-analysis. Sci Rep.

[30] Beck AT, Clark DA (2011). Cognitive Therapy of Anxiety Disorders: Science and Practice.

[31] de Neef M, Cuijpers P (2016). Fobieën.

[32] De Jong PJ, Keijsers GPJ (2011). Protocollaire behandelingen voor volwassenen met psychische stoornissen.

[33] Beck A, Epstein N, Brown G, Steer RA (1988). Beck anxiety inventory (BAI). Überblick über Reliabilitäts-und Validitätsbefunde von klinischen und außerklinischen Selbst-und Fremdbeurteilungsverfahren. J Consult Clin Psychol.

[34] Brown GK, Beck AT, Newman CF, Beck JS, Tran GQ (1997). A comparison of focused and standard cognitive therapy for panic disorder. J Anxiety Disord.

[35] Beck A, Steer R (2015). BAI-NL Beck Anxiety Inventory - Nederlandse versie. Pearson.

[36] Patient Health Questionnaire (PHQ) Screeners. Pfizer.

[37] Kroenke K, Spitzer RL, Williams JBW (2001). The PHQ-9: validity of a brief depression severity measure. J Gen Intern Med.

[38] Wittkampf KA, Naeije L, Schene AH, Huyser J, van Weert HC (2007). Diagnostic accuracy of the mood module of the Patient Health Questionnaire: a systematic review. Gen Hosp Psychiatry.

[39] Donker T, van Straten A, Marks I, Cuijpers P (2009). A brief Web-based screening questionnaire for common mental disorders: development and validation. J Med Internet Res.

[40] Devilly GJ, Borkovec TD (2000). Psychometric properties of the credibility/expectancy questionnaire. J Behav Ther Exp Psychiatry.

[41] Mertens VC, Moser A, Verbunt J, Smeets R, Goossens M (2017). Content validity of the Credibility and Expectancy Questionnaire in a pain rehabilitation setting. Pain Pract.

[42] Attkisson C, Greenfield T, Maruish ME (2004). The use of psychological testing for treatment planning and outcomes assessment: Instruments for adults. The Client Satisfaction Questionnaire-The UCSF Client Satisfaction Scales: I.

[43] de Brey H (1983). A cross-national validation of the client satisfaction questionnaire: the Dutch experience. Eval Program Plann.

[44] Bangor A, Kortum PT, Miller JT (2008). An empirical evaluation of the System Usability Scale. Int J Hum Comput Interact.

[45] Sauro J (2011). A Practical Guide to the System Usability Scale: Background, Benchmarks & Best Practices.

[46] Davis M (1983). Measuring individual differences in empathy: Evidence for a multidimensional approach. J Pers Soc Psychol.

[47] De Corte K, Buysse A, Verhofstadt LL, Roeyers H, Ponnet K, Davis MH (2007). Measuring empathic tendencies: Reliability and validity of the Dutch version of the Interpersonal Reactivity Index. Psychol Belg.

[48] Cornelisz I, Cuijpers P, Donker T, van Klaveren C (2020). Addressing missing data in randomized clinical trials: A causal inference perspective. PLoS One.

[49] Bouchard S, Côté S, St-Jacques J, Robillard G, Renaud P (2006). Effectiveness of virtual reality exposure in the treatment of arachnophobia using 3D games. Technol Health Care.

[50] Emmelkamp PMG, Krijn M, Hulsbosch AM, de Vries S, Schuemie MJ, van der Mast CAPG (2002). Virtual reality treatment versus exposure in vivo: a comparative evaluation in acrophobia. Behav Res Ther.

[51] Piercey CD, Charlton K, Callewaert C (2012). Reducing anxiety using self-help virtual reality cognitive behavioral therapy. Games Health J.

