# Supplementary material for: Virtual Reality Self-help Treatment for Aviophobia: Protocol for a Randomized Controlled Trial
Source: JMIR Res Protoc. 2021 Apr 12;10(4):e22008. doi: 10.2196/22008 (PMC8076993; doi:10.2196/22008)
Supplement: Multimedia Appendix 1 [file resprot_v10i4e22008_app1.pdf]

	STUDY PERIOD					
	**Enrollment**	**Allocation**	**Test Phase**	**Close-out**		
**TIMEPOINT**	***$-t_1$***	**0**	***6 weeks***	***t_1***	***t_2***	***t_3***
**ENROLLMENT:**						
**Eligibility screen**	X					
**Informed consent**	X					
Baseline		X				
**Allocation**		X				
**INTERVENTIONS:**						
Treatment			X	X	X	X
Waitlist Control			X	X		
**ASSESSMENTS:**						
FAS	X			X	X	X
FAM		X		X	X	X
BAI		X		X	X	X
PHQ-9		X		X	X	X
WSQ		X				
IRI		X				
Flight usage questions		X		X	X	X
CEQ		X				
SUS				X*		
IPQ				X*		
CSQ				X*		
Ecological momentary assessment			X			
